# La Parasitología en Colombia: una visión panorámica

**Published:** 2021-05-31

**Authors:** Sofía Duque, Adriana Arévalo, Rubén Santiago Nicholls

**Affiliations:** 1 Grupo de Parasitología, Instituto Nacional de Salud, Bogotá, D.C., Colombia sduque@ins.gov.co Grupo de Parasitología Instituto Nacional de Salud BogotáD.C Colombia sduque@ins.gov.co; 2 Grupo de Parasitología, Instituto Nacional de Salud, Bogotá, D.C., Colombia aarevalo@ins.gov.co Grupo de Parasitología Instituto Nacional de Salud BogotáD.C Colombia aarevalo@ins.gov.co; 3 Investigador Emérito, Instituto Nacional de Salud, Bogotá, D.C., Colombia rsnichollso@gmail.com Instituto Nacional de Salud BogotáD.C Colombia

«Serré, fourmillant, comme un million d'helminthes, dans nos cerveaux ribote un peuple de Démons, et, quand nous respirons, la Mort dans nos poumons descend, fleuve invisible, avec de sourdes plaintes». *Au lecteur, Les fburs du mal,* Charies Baudelaire (1821-1867)

«Oprimido, hormigueante, como un millón de helmintos, en nuestros cerebros bulle un pueblo de Demonios, y, cuando respiramos, la Muerte a los pulmones desciende, río invisible, con sordas quejas». *Al lector, Las fbres del mal,* Charles Baudelaire (1821-1867)

En Colombia, las enfermedades tropicales endémicas, entre ellas las parasitosis humanas, han sido de interés como problemas de salud pública y como objeto de investigación desde comienzos del siglo XX hasta la fecha. El Instituto Nacional de Salud fue una de las instituciones pioneras en este campo, al desarrollar investigaciones y generar conocimiento científico, lo cual ha contribuido a comprender mejor la epidemiología de las enfermedades parasitarias en el país y a mejorar sus programas de control.

Entre los científicos colombianos que pueden ser considerados como precursores die la Parasitología en Colombia, se encuentran nombres ilustres que dejaron su huella en el Instituto, como César Uribe Piedrahita, Santiago Renjifo, Hernando Groot, Ernesto Osorno Mesa, Augusto Gast Galvis, Carlos Sanmartín Barberi y Augusto Corredor Arjona. Entre sus muchas contribuciones cabe destacar la demostración por primera vez de la existencia de *Trypanopoma cruzi* y *Trypanrsomc rangeli* en Colombia ^1^, el reporte del primer caso de leishmaniasis visceral en el país ^2^, el estudio de t_r_ipanosomas humanos y de animales ^3^, el primea estudio sobre la distribución de los triatominos domiciliarios vectores de la enfermedad de Chagas ^4^, *y* las primeras encuestas nacionales de prevalencia de toxoplasmosis ^5^ y de parasitismo intestinal ^6^.

En la segunda mitad del siglo XX, particularmente a partir de la década de 1960, surgieron en el país centros e institutos de investigación en Parasitología y Medicina Tropical vinculados a instituciones o a universidades públicas o privadas, como el Laboratorio de Parasitología del Instituto Nacional de Salud, el Centro de Investigaciones en Microbiología y Parasitología "Tropical de la Universidad de los Andes, el Centro de Investigaciones en Enfermedades Tropicales de la Universidad Industrial de Santander, el Centro internacional de Entrenamiento e Investigaciones Médicas, el Instituto Colombiano de Medicina Troncal, la Corporación para Investigaciones Biológicas, el Programa para el Estudio y Control de las Enfermedades Tropicales de la Universidad de Antioquia, el Laboratorio de Investigaciones en Parasitología Tropical de la Universidad del Tolima y el Grupo de Investigación en Parasitología Molecular de la Universidad del Quindío, entre otros. Por otro lado, en 1965, se fundó la Asociación Colombiana de Parasitología y Medicina Tropical que congrega a los investigadores de esta área del conocimiento de estas organizaciones.

Todos estos centros, que gozan de reconocimiento nacional e internacional, han contribuido a la comprensión de las parasitosis, al conocimiento de sus agentes etiológicos, su ciclo de vida, su transmisión, su diagnóstico parasitológico, serológico y por detección de antígenos mediante diferentes metodologías que han evolucionado a lo largo del tiempo, desde los métodos parasitológicos hasta incorporar las aplicaciones de la biología molecular, la proteómica y la genómica. Asimismo, han enriquecido el conocimiento de sus manifestaciones clínicas, su terapéutica, los aspectos epidemiológicos, los factores de riesgo, y las intervenciones de prevención y control.

Las alianzas interdisciplinarias dentro de las instituciones y entre ellas -a nivel nacional e internacional- han permitido el desarrollo de la Parasitología y el entendimiento de los parásitos desde diferentes puntos de vista científicos, así como una aproximación integral a las parasitosis. Cabe destacar que los centros de investigación han colaborado con el Ministerio de Salud y Protección Social, así como con las autoridades departamentales y municipales de salud, para fortalecer los programas de control y eliminación de las parasitosis humanas, con el propósito de beneficiar a las poblaciones expuestas y vulnerables.

Las enfermedades parasitarias están íntimamente relacionadas con la pobreza y las deficientes condiciones de vida, como la falta de acceso al agua potable, al saneamiento básico, a la vivienda adecuada y a la educación. La mayoría de ellas se clasifican entre las enfermedades tropicales desatendidas y perpetúan el círculo vicioso de pobreza y enfermedad, porque contribuyen a disminuir la capacidad productiva y los ingresos de las personas afectadas y de sus familias. En algunos casos, como en los de las geohelmintiasis, la enfermedad de Chagas y la toxoplasmosis, las consecuencias crónicas de la infección durante la infancia se manifiestan en la adolescencia o en la edad adulta. Es evidente la relación de la malaria con algunas actividades ilícitas que se ejercen en ambientes selváticos, como la minería ilegal o el cultivo y procesamiento de la hoja de coca. El conflicto armado contribuyó al aumento de casos y a la aparición de brotes de algunas de estas enfermedades, como la leishmaniasis cutánea.

La recesión, el desempleo y el aumento de la pobreza -desastrosas consecuencias sociales y económicas de la pandemia del Covid-19- han deteriorado aún más las condiciones de vida de las poblaciones vulnerables y, en consecuencia, puede darse a mediano plazo un aumento de la incidencia y la prevalencia de las parasitosis endémicas.

El control o la eliminación de las enfermedades parasitarias y desatendidas requieren de un manejo integral que incluya los factores sociales y ambientales determinantes de la salud. Las ciencias de la Parasitología clásica y molecular deben complementarse, no solamente con otras ciencias biológicas y de la salud, sino también con las ciencias humanas y sociales. Solo así es posible lograr un control sostenible de las parasitosis endémicas que, además, contribuya a mejorar el nivel de vida de las poblaciones bajo riesgo.

Como todos los países miembros de la Organización de las Naciones Unidas, Colombia está comprometida con el logro de los objetivos de desarrollo sostenible para el 2030. El objetivo 3, "Garantizar una vida sana y promover el bienestar para todos en todas las edades" ^7^, incluye como una de sus metas la eliminación de la malaria y las enfermedades tropicales desatendidas. Siendo conscientes del impacto negativo que la pandemia ha tenido sobre los programas de control de las enfermedades parasitarias, una vez superada esta, uno de los mayores desafíos será el de recuperar el tiempo y los avances perdidos e intensificar las intervenciones para alcanzar la meta del objetivo 3 en el 2030.
